# Nocardiosis in a renal transplant recipient following rituximab preconditioning

**DOI:** 10.1080/03009730802604931

**Published:** 2009-02-04

**Authors:** Tanya R Flohr, Costi D Sifri, Kenneth L Brayman, Klaus D Hagspiel, Robert G Sawyer, Timothy L Pruett, Hugo JR Bonatti

Sir,

With great interest we read the recent article ‘Pulmonary nocardiosis with brain abscess in a sensitized kidney transplant recipient with history of repeated graft loss and human leukocyte antigen HLA-antibody depletion treatment—a case report’ by Ali-Reza Biglarnia et al. (1). This report is important in the light of an increased exposure of renal transplant recipients to more intense immunosuppression, in particular to depleting antibodies such as rituximab. Rituximab is unique, as in contrast to antithymocyte globulin (ATG), muromunab OKT3, and alemtuzumab it targets B cells (2) but may cause profound hypogammaglobulinemia. We would like to add another case of nocardiosis after renal transplantation (RT) and exposure to rituximab that largely mimics the case reported by Biglarnia and should support the findings and emphasize the risk for opportunistic infections in such patients.

Our patient was a 45-year-old female with end stage renal disease secondary to streptococcal glomerulonephritis. She underwent living related RT in 1978 but ceased taking her immunosuppression during the early 1990s for an unknown period. In 1997, she presented with an elevation in her serum creatinine from 1.5 to 5.0 mg/dL. This non-compliance-associated rejection was initially treated with a course of methylprednisolone (500 mg daily for three doses), then with a course of OKT3 (5 mg daily for five days). The rejection could not be reversed, and she was started on hemodialysis in 1998. The patient tested repeatedly positive for preformed panel-reactive antibodies (PRA) with high titers, and on several occasions the donor/recipient cross-match was positive, preventing a cadaveric RT. The option for living related donation was evaluated; however, the cross-match was again incompatible. Therefore, the patient underwent preconditioning for RT with rituximab (375 mg/m^2^), intravenous immunoglobulin (IVIG) at a dose of 0.5–4.0 mg/kg/min, and whole plasma exchange (WPE). During the waiting time for RT she was maintained on mycophenolate mofetil (MMF) at a dose of 2 g/day. Her subsequent titer of PRAs was found to be suitable to proceed with RT. Living related RT from a cousin was performed without complication in 2006, and immunosuppression consisted of ATG induction (1.5 mg/kg for three days) followed by tacrolimus (TAC) with trough levels of 6–12 ng/mL, with continuation of MMF and a steroid taper. Two weeks post RT, a biopsy revealed humoral rejection. She was treated with ATG, IVIG, and nine courses of WPE. Rejection resolved, and she was discharged with stable graft function. *Pneumocystis jirovecii* prophylaxis consisted of dapsone due to her sulfa allergy; She was hospitalized multiple times for anasarca, urinary tract infection, and pneumonia. Despite several attempts to counsel the patient, she continued smoking and missed several follow-up appointments.

**Figure 1.**
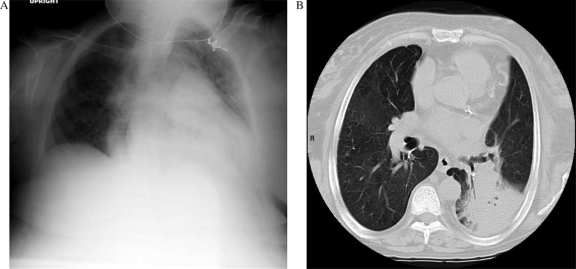
Chest X-ray (A) and CT scan (B) show left lobe infiltrates.

Three months post RT she developed another episode of pneumonia presenting with shortness of breath, cough, and fever. Chest X-ray revealed a left upper lobe lesion consistent with infection in the context of the clinical history. No pathogen could be isolated from sputum. She received a 10-day course of empiric therapy with linezolid (600 mg daily) and ciprofloxacin (500 mg twice daily), and immunosuppression was temporarily reduced. She rapidly improved and was discharged with stable graft function. Two months later, she again deteriorated; her left upper lobe lesion appeared unresolved and cavitary on chest X-ray and CT scan (Figure 1). Bronchoalveolar lavage cultures revealed *Nocardia nova*. She was desensitized and started on twice daily double-strength trimethoprim/sulfamethoxazole (TMPS); immunosuppression was again temporarily reduced. She was discharged with resolved respiratory symptoms and stable graft function, however, again deteriorated requiring readmission six months after first diagnosis of nocardiosis. She developed respiratory failure requiring mechanical ventilation. *Nocardia* could not be isolated, however, TMPS was continued. She again improved and was discharged with stable graft function. Subsequently, she was hospitalized another two times for respiratory infections. Her chest CT scan continued to reveal a lung lesion with slow resolution. She is currently alive with good graft function, no further rejection episodes and no signs of recurrent nocardiosis.

Similar to the reported case by Biglarnia et al. our patient had a re-RT, and the first RT was complicated by steroid-resistant rejection and OKT3 treatment. In addition both patients had elevated PRA and underwent preconditioning using WPE and rituximab. Both received TAC, MMF, and steroids for maintenance immunosuppression. Our patient may have received even more intense immunosuppression (ATG versus interleukin IL-2 receptor antagonist induction), and in addition she had rejection requiring a second course of ATG and WPE. On the other hand our patient was 14 years younger. Both patients had no prophylaxis against *Nocardia* as their *Pneumocystis jirovecii* prophylaxis included dapsone and inhaled pentamidine. Recent findings by the Pittsburgh group suggested that in a subset of patients, TMPS may be ineffective in terms of prophylaxis against *Nocardia* (3). The application of linezolid for pneumonia may have temporarily controlled *Nocardia* growth. This new agent may be a good treatment option in transplant recipients, and one patient in the publication from the Innsbruck group was successfully treated with linezolid (3). In our patient, we assume that the continuous smoking was a significant contributor. Nocardiosis has been reported in a RT recipient who was a smoker (4). We have recently encountered an increasing number of transplant recipients with nocardiosis. In addition, extrapulmonary manifestations have become more common, and species other than *Nocardia asteroides* are on the rise. Our patient had only pulmonary involvement in contrast to the previously reported case, and in both cases non-asteroides *Nocardia* were the causing organism (*Nocardia nova* and *Nocardia farcinica*). A common denominator in 11 transplant recipients who developed nocardiosis between 1995 and 2008 at our center was the use of depleting antibodies. Transplant physicians should be aware of this rare infection and include nocardiosis in the differential diagnosis in patients presenting with pneumonia.
